# Ameliorative Potential of *Tamarindus indica* on High Fat Diet Induced Nonalcoholic Fatty Liver Disease in Rats

**DOI:** 10.1155/2014/507197

**Published:** 2014-02-04

**Authors:** Suja Rani Sasidharan, Joshua Allan Joseph, Senthilkumar Anandakumar, Vijayabalaji Venkatesan, Chandrasekharan Nair Ariyattu Madhavan, Amit Agarwal

**Affiliations:** ^1^College of Veterinary and Animal Sciences, Mannuthy, Thrissur 680 651, India; ^2^R&D Centre, Natural Remedies, Plot No.5B, VeerasandraIndl. Area, 19th K.M. Stone, Hosur road, Electronic city, Bangalore, Karnataka 560 100, India

## Abstract

Nonalcoholic fatty liver disease (NAFLD), the prevalence of which is rising globally with current upsurge in obesity, is one of the most frequent causes of chronic liver diseases. The present study evaluated the ameliorative effect of extract of *Tamarindus indica* seed coat (ETS) on high fat diet (HFD) induced NAFLD, after daily administration at 45, 90, and 180 mg/kg body weight dose levels for a period of 6 weeks, in albino Wistar rats. Treatment with ETS at all tested dose levels significantly attenuated the pathological alterations associated with HFD induced NAFLD *viz*. hepatomegaly, elevated hepatic lipid and lipid peroxides, serum alanine aminotransferase, and free fatty acid levels as well as micro-/macrohepatic steatosis. Moreover, extract treatment markedly reduced body weight and adiposity along with an improvement in insulin resistance index. The study findings, therefore suggested the therapeutic potential of ETS against NAFLD, acting in part through antiobesity, insulin sensitizing, and antioxidant mechanisms.

## 1. Introduction

Nonalcoholic fatty liver disease (NAFLD) refers to the spectrum of pathological conditions characterized by fatty infiltration of the liver, ranging from simple steatosis through nonalcoholic steatohepatitis (NASH) to fibrosis and cirrhosis that occur in absence of alcohol consumption, viral infection, or other specific etiologies [1]. Because of its strong association with obesity, reduced glucose tolerance, type 2 diabetes mellitus, arterial hypertension, and hypertriglyceridaemia, NAFLD is now universally considered as the hepatic manifestation of the metabolic syndrome with insulin resistance as the key pathophysiological hallmark. The prevalence of NAFLD has reached epidemic proportions in recent years, on a par with the increasing prevalence of obesity and metabolic syndrome worldwide, and is the most common source of abnormal liver function tests and chronic liver diseases in both developed and developing countries [2]. It has been estimated recently that 10–24% of the world's population suffer from the condition and its prevalence reached almost 60–95% and 28–55% in obese and diabetic patients, respectively [3]. Thus, in light of these data, there is a critical need to elucidate the mechanisms that mediate the development and progression of NAFLD and to identify potential therapies for the disease.

The molecular mechanism for the initiation and progression of NAFLD remains poorly understood. According to the traditional “two-hit” pathophysiological theory, hepatic fat accumulation is purported to be the “first hit” and the prerequisite for hepatocyte injury to develop, whereas mitochondrial fatty acid oxidation that produce reactive oxygen species (ROS) inducing intracellular oxidative stress, inflammation, and gross injuries to hepatocytes and eventually culminating in fibrosis represented the “second hit” for the progression to NASH. However, the “two-hit” hypothesis was recently questioned and is now being modified by the “multiple parallel hits” hypothesis for explaining the progression from NAFLD to NASH. In the “multiple-hit” model, insulin resistance and the associated disrupted lipid metabolism is the first step towards NAFLD development. After the initial hepatic infiltration, the liver becomes extremely vulnerable to hyperinsulinemia-induced “multihit” events, which include oxidative damage, dysregulated hepatocyte apoptosis, activation of the profibrogenic factors and proinflammatory mediators, dysregulation of multiple adipokines, and hepatic stellate cell activation. The cascade of events induces excessive accumulation of extracellular matrix proteins, such as collagen, to cause fibrosis with concomitant necroinflammation and cell death [2, 4].

However, an approved standardized treatment for NAFLD is lacking till date, despite its high prevalence and severe challenge to healthcare systems. The current strategies to inhibit NAFLD and NASH progression consist of weight management, adipose tissue and oxidative stress reduction, improvement in insulin resistance, and lipid profile optimization. Insulin receptor sensitizing agents and antioxidants have been tested for the treatment of NAFLD, albeit their clinical efficacy and safety remain to be established [5, 6]. Moreover, it is a major challenge for pharmaceutical industry to develop a combination therapy that is effective against NAFLD patients with obesity, insulin resistance, dyslipidemia, and oxidative stress. Therefore, earnest efforts have been directed presently at exploring new therapeutic agents that can be aimed at multiple targets. Numerous studies have suggested that natural constituents in food, especially polyphenolic phytoprinciples, namely, flavonoids and tannic acid, are ubiquitous in plants with insulin sensitizing, hypolipidemic and antioxidant potentials and are easily accessible to man through their diets [7]. Herbal medicines, comprising of innumerable phyto-principles, hence are becoming increasingly popular and targeted for the effective management of NAFLD, because of their minimal side effects and their multiple mode of action in controlling lipid metabolism [8].

*Tamarindus indica *Linn., belonging to the Caesalpiniaceae family, grows naturally in tropical and subtropical regions and is now one of the most important plant resources as food materials and is also accepted as herbal medicine in different parts of the world. Various parts of *T. indica* have been reported for a multitude of health benefits such as antioxidant, antihepatotoxic, antiinflammatory, antimutagenic, carminative, and expectorant properties [9]. However, the therapeutic potential of *T. indica* seed coat remains largely unexplored, although free radical scavenging activity and total phenolics content of tamarind seed coat extract have been demonstrated in various studies [9, 10]. Pumthong [11] reported that tamarind seed coat is composed of polyphenols including tannins, anthocyanidin, and oligomericanthocyanidins. Keeping these facts in concern, the present study was designed to evaluate the ameliorative potential of extract of *T. indica* seed coat (ETS) on HFD induced NAFLD in rats.

## 2. Materials and Methods

### 2.1. Animals and Diet

Male albino Wistar rats (160–180 g body weight) of the present study were acclimatized for a week before experimentation and maintained at optimal temperature with 12 hlight/dark cycle and 30–70% relative humidity. The animals were provided either with customized semipurified normal control diet (ND, modified AIN-93G diet with 7.1% kcal fat) or high fat diet (HFD, modified AIN-93G diet with 45% kcal fat) and U.V. purified water, *ad libitum*. The composition (Table 1) and preparation of the diets has been described in our earlier report [12]. The experimental protocol was approved by the Institutional Animal Ethics Committee of College of Veterinary and Animal Sciences, Kerala Agricultural University.

### 2.2. Preparation of Extract of *T. indica* Seed Coat

Tamarind seed coat was procured from commercial suppliers and were identified and authenticated at the National Institute of Science Communication and Information Resources (NISCAIR), New Delhi. The ground tamarindus seed coat was initially extracted with methanol in 1 : 4 ratio for 1 hour at 60–65°C. The liquid extract attained was filtered and the procedure was repeated twice with marc using methanol (1 : 3). The thick extract thus obtained was dried under vacuum (at ≤70°C) and was further sieved to get the final methanolic extract, which on further refluxing with water (1 : 4) at 80–90°C for 2 hours was kept at 2–5°C for 24 hours in a well-closed container. The sediment formed at bottom of the container was removed while liquid supernatant obtained was concentrated under reduced pressure, filtered to get clear liquid, and further spray dried.

### 2.3. Experimental Design

Male albino Wistar rats used in the experiment were divided into two dietary regimen groups by feeding either ND or HFD for the initial 6 weeks of induction period. Five animals from each group were sacrificed and liver was collected to estimate hepatic total lipid, triglycerides, and cholesterol to confirm the induction of fatty liver. Consequently, six animals from the ND fed rats were allotted to Group I (normal diet control, ND control) and were administered with vehicle (distilled water at 10 mL/kg b.w.). Twenty-four HFD fed rats were randomly assigned into four groups (Group II to V), of six animals each. Group II served as NAFLD control (distilled water at 10 mL/kg b.wt), while groups III to V were treated with ETS at the dose levels of 45, 90, and 180 mg/kg b.w., respectively. All the treatments were given daily by oral gavage during the subsequent treatment period of 6 weeks.

### 2.4. Measurement of Bodyweight and Adiposity

During the study, body weight was recorded on days 0 and 42 of induction period and further at fortnightly intervals during the six-week treatment period. At the end of the experiment, adipose depots such as epididymal, perirenal, retroperitoneal, and mesenteric fats were collected after euthanasia and their relative weights were recorded. Adiposity index was computed as sum of the fat pads/(body weight − fat pad weight) × 100 [13].

### 2.5. Measurement of Hepatic Parameters

The rat livers were excised, rinsed with cold phosphate-buffered saline, and then weighed to record relative liver weight/liver index. The level of total liver lipid was estimated using the method described by Cho [14] and the concentrations of triglyceride and cholesterol were measured using commercial enzyme assay kits (M/s Span Diagnostics Ltd., India). Level of hepatic lipid peroxides in tissue homogenate was determined by the method of Lee et al. [15]. The representative samples of liver tissue were stored in 10% buffered neutral formalin and were processed for histological examination. Sections were cut and stained with hematoxylin and eosin (H&E).

### 2.6. Measurement of Serum Parameters

Serum free fatty acid (FFA), glucose, and alanine aminotransferase (ALT) were measured using commercial enzyme assay kits (Wako Pure Chemicals, Japan and Span Diagnostics Ltd., India) and insulin was estimated by homogeneous time resolved fluorescence (HTRF) Sandwich assay using commercial insulin kit (Cisbio International, France). Insulin resistance index such as homeostatic model assessment (HOMA) was computed using the below formula [16]:

(1) HOMA =fasting  insulin  (μIU/mL)×fasting  glucose  (mmol/mL)22.5.

### 2.7. Statistical Analysis

All values are expressed as mean ± SEM and statistical analysis was carried out using statistical package for social science (SPSS). The data were analyzed using one-way ANOVA followed by Bonferroni's post hoc test for multiple group comparison. Student's *t*-test was carried out to determine the significant difference in parameters of studies with ND and HFD treatment groups alone. Statistical significance was set at *P* ≤ 0.05.

## 3. Results

### 3.1. Effect of ETS on Bodyweight and Adiposity

**Figure 1:**
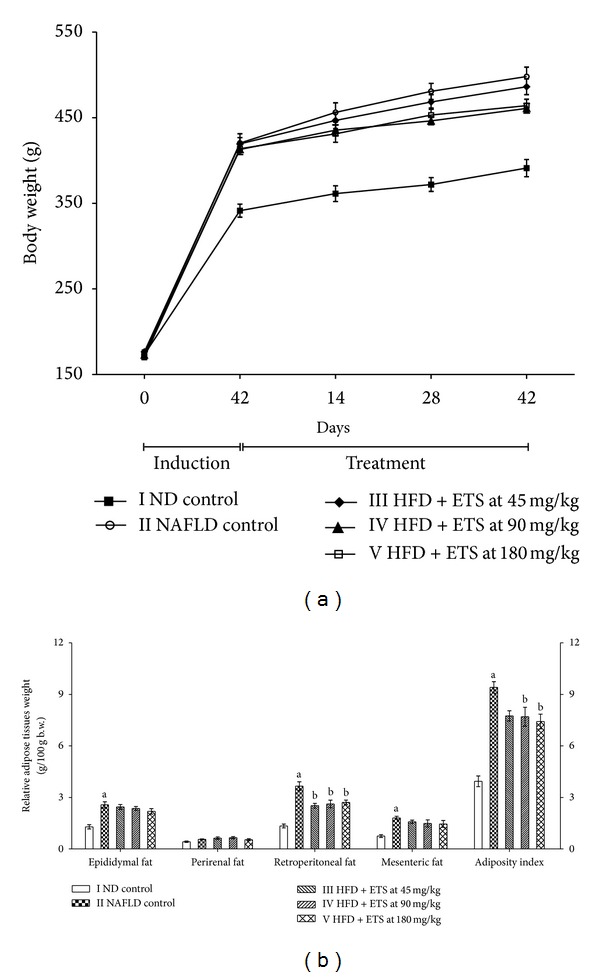
Effect of ETS treatment on body weight and relative adipose tissue weights in high fat diet fed rats. (a) Body weights; (b) relative adipose tissue weights. Rats were orally treated by high fat diet with or without ETS daily for 6 weeks, after induction of NAFLD by HFD feeding for 6 weeks. Values are expressed as Mean ± SEM; *n* = 6; ^a^ *P* ≤ 0.05, NAFLD control vs ND control, ^b^ *P* ≤ 0.05, treated groups vs NAFLD control ETS: extract of *Tamarindus indica *seed coat; ND: normal diet; HFD: high fat diet; NAFLD: nonalcoholic fatty liver disease.

The rats fed with HFD showed significantly (*P* ≤ 0.05) increased body weight at the end of the induction period and the HFD fed NAFLD control rats continued the significant (*P* ≤ 0.05) increase throughout the treatment period as compared to ND control group. Treatment with ETS at 90 and 180 mg/kg showed an evident reduction in the body weight in HFD fed rats throughout the treatment period, whereas ETS at 90 mg/kg offered even a significant reduction on 28th day of treatment (Figure 1(a)). The relative weights of epididymal, retroperitoneal, and mesenteric fats and the adiposity indices were found to be significantly (*P* ≤ 0.05) increased in NAFLD control rats as compared to ND control group, while administration of ETS, at all dose levels, significantly (*P* ≤ 0.05) decreased the relative weights of retroperitoneal fat as well as adiposity index, except for a nonsignificant reduction in adiposity index observed at 45 mg/kg (Figure 1(b)).

### 3.2. Effect of ETS on Hepatic Parameters

**Figure 2:**
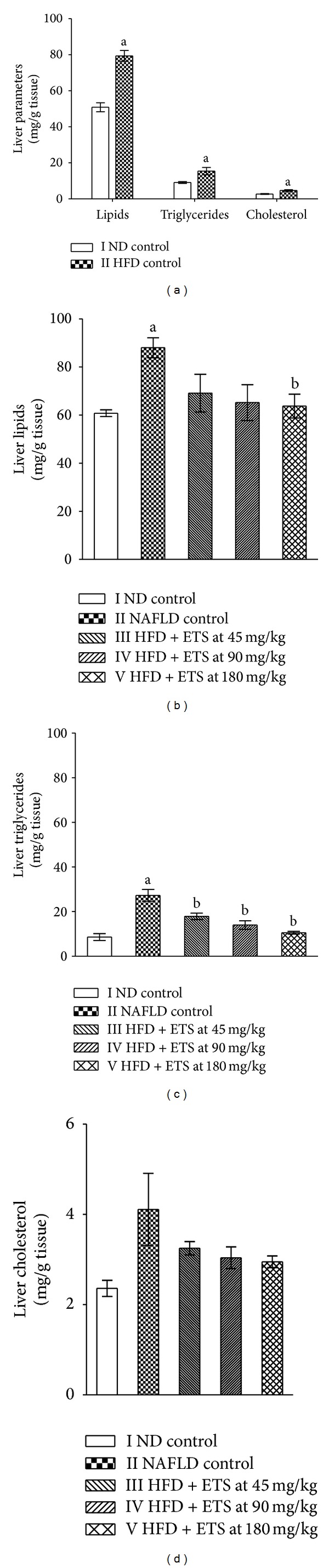
Effect of high fat diet and ETS treatment on liver lipid parameters in rats (a) after induction (b) after ETS treatment. Rats were orally treated by high fat diet with or without ETS daily for 6 weeks, after induction of NAFLD by HFD feeding for 6 weeks. Values are expressed as Mean ± SEM; *n* = 6; ^a^ *P* ≤ 0.05, HFD/NAFLD control vs ND control, ^b^ *P* ≤ 0.05, treated groups vs NAFLD control ETS: extract of *Tamarindus indica *seed coat; ND: normal diet; HFD: high fat diet; NAFLD: nonalcoholic fatty liver disease.

HFD fed control rats showed a significant increase (*P* ≤ 0.05) in hepatic lipids, triglycerides and cholesterol in comparison to ND fed rats after six weeks of induction (Figure 2(a)). NAFLD control rats exhibited a significant (*P* ≤ 0.05) increase in total hepatic lipids, triglycerides with a nonsignificant increase in hepatic cholesterol levels as compared to ND control group rats after six weeks of treatment period as well. Nevertheless, ETS treatment at all dose levels exhibited a significant (*P* ≤ 0.05) reduction in hepatic triglycerides and a nonsignificant decrease in hepatic cholesterol levels. Moreover, ETS, decreased hepatic total lipids significantly at 180 mg/kg whilst nonsignificantly at 45 and 90 mg/kg (Figures 2(b), 2(c), and 2(d)). Meanwhile, relative liver weight/liver index in the NAFLD control group was significantly higher than that of ND control group (*P* ≤ 0.05), while ETS treatment at all the dose levels had a significantly lower liver index than the NAFLD control group (*P* ≤ 0.05). Considering hepatic lipid peroxides indicated as MDA, the levels in HFD fed NAFLD control rats was found to be significantly higher when compared to normal control rats, whereas administration of ETS, at all dose levels, exhibited a significant (*P* ≤ 0.05) reduction in hepatic lipid peroxides levels in HFD fed rats (Table 2).

**Figure 3:**
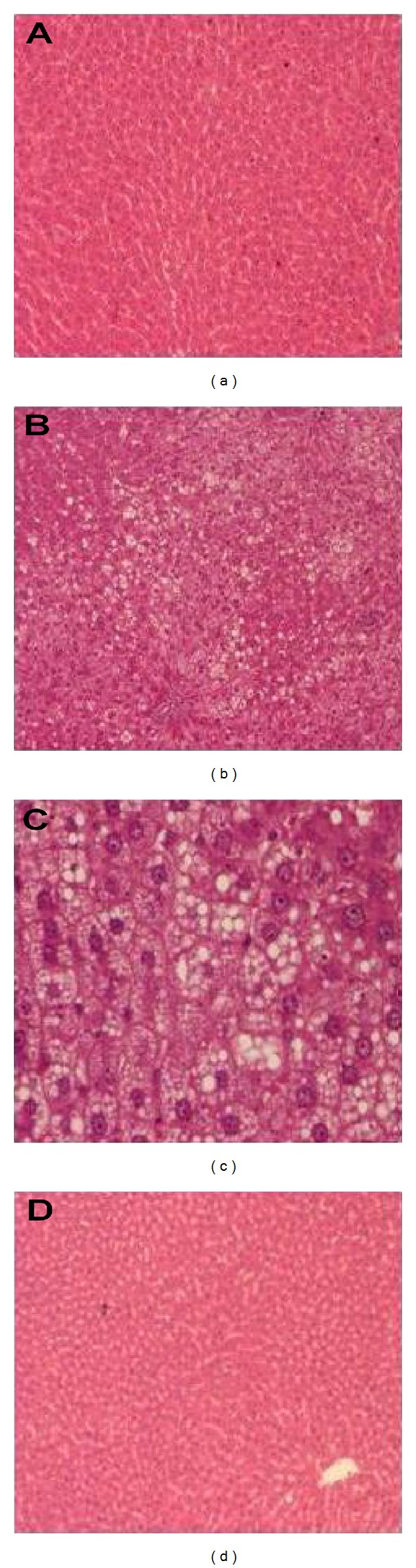
Histology of hepatic tissue sections dyed with haematoxylin-eosin (HE). (a) ND control (100x) showing normal structure and architecture (b) HFD fed NAFLD control (100x) and (c) HFD fed NAFLD control (400x) showing severe degree of micro and macro vescicular steatosis and severe hepatocellular ballooning (d) HFD fed ETS treated at 90 mg/kg body weight group (100x) showing near normal structure. ETS: extract of *Tamarindus indica *seed coat; ND: normal diet; HFD: high fat diet; NAFLD: nonalcoholic fatty liver disease.

The histological examination of the liver section from ND control group showed normal structure and architecture, while NAFLD control rats showed moderate to severe degree of micro- and macrovesicularsteatosis, hepatocellular ballooning, vacuolar degeneration with ground glass appearance, and moderate biliary hyperplasia. The severity of degenerative changes was narrowed by *T. indica* extract treatment when compared to the NAFLD control group and showed only a very mild mononuclear cell infiltration around portal triad. Liver section of few of the ETS treated animals revealed even a near normal architecture (Figure 3).

### 3.3. Effect of ETS on Serum Parameters

The NAFLD control rats showed a significant (*P* ≤ 0.05) increase in serum ALT, FFA, and insulin levels along with HOMA index, in consort with a nonsignificant increase in serum glucose, as compared to ND control rats. However, ETS treatment, at all dose levels, exhibited significant (*P* ≤ 0.05) reduction in serum ALT and FFA when compared to NAFLD control rats. Besides, ETS decreased serum glucose significantly at 180 mg/kg b.w. and nonsignificantly at other dose levels. A nonsignificant decrease in serum insulin level and HOMA index was observed in ETS treated HFD fed rats at all dose levels (Table 2).

## 4. Discussion

Liver plays a vital role in lipid metabolism; its role includes uptake of FFA (released from both diet and adipose tissues) and *de novo* synthesis of FFA followed by its conversion into triglycerides by esterification. The triglycerides are then released into the circulation as very low-density lipoproteins (VLDL) or stored as triglyceride vacuoles in hepatocytes. FFA that are not esterified into triglycerides will be metabolized in the liver by *β*-oxidation. In brief, NAFLD is a condition wherein a disruption of this cascade of events occurs as the amount of FFA delivered or synthesized in the liver exceeds its oxidative capacity and leads to increased triglyceride retention. Consequently, triglyceride synthesis continues to rise and exceed the amount that can be released as VLDL, paving way to triglyceride accumulation in hepatocytes causing hepatic steatosis [17].

Animal models of NAFLD may be divided into two broad categories: those caused by genetic mutation and those with an acquired phenotype produced by dietary or pharmacological modification. Common rodent models including leptin deficient genetic models or a diet deficient in choline and methionine have hitches in mimicking the initiation and progression of human NAFLD, because caloric overconsumption is a major factor in NAFLD development in the clinical condition. Hence, recent studies have often explored the possibility of using HFD to induce a similar condition in rats [18]. Excessive dietary fat can result in increased FFA levels in the blood, thereby amplifying the delivery of FFA to the liver. Undue consumption of dietary fats thus induces lipid accumulation in the liver and can also precipitate obesity and associated metabolic disorders, resembling human metabolic syndrome [19]. Lieber et al. [20] found that a highfat, highcalorie liquid diet (71% energy from fat) induced steatosis effectively, while Svegliati-Baroni et al. [21] used a commercially available solid highfat diet (58% energy from fat) and reported to cause the common features, not only of NAFLD and NASH but also obesity, insulin resistance, oxidative stress, and inflammation. In our study, we used a customized, semipurified HFD of 45% energy from fat, formulated based on AIN-93 rodent diet composition with minor modifications and lard as major fat component Furthermore, the fat composition of HFD in the present study is closer to the clinical fat consumption level of about 30% [22] when compared to other HFD models (58–71%), thus better suited physiologically for studying the basic pathogenesis of NAFLD.

By means of the present HFD fed rat model, we have been able to demonstrate most of the clinical aspects of NAFLD in humans such as increased hepatic lipids accompanied by different degrees of steatosis histologically, elevated serum ALT, increase in body weight and adiposity, elevated serum FFA, increased HOMA-IR index, and elevated hepatic lipid peroxides level. These findings are in quite corroboration with previous reports [19, 21] and thus depicted effective establishment of NAFLD in HFD fed control rats, indicating the suitability of the present NAFLD model for screening and evaluation of test substances for the treatment of NAFLD. The prime outcome of the present study was that the extract of *Tamarindus indica* (ETS) ameliorates not only hepatic steatosis and degeneration but also other allied clinical features of NAFLD such as visceral obesity, altered lipid metabolism, insulin resistance, and oxidative stress in HFD induced rat model of NAFLD.

HFD feeding precipitates the characterization of obesity development that causes increase in body weight with expanded adipose mass and may well lead to altered function of adipocytes [19]. This paves way to increased insulin secretion, increased release of FFA in the blood, and finally increases the amount of triglyceride storage in the liver. The surplus storage of triglyceride in liver in due course develops into larger and more abundant lipid accumulations, resulting in fatty liver. Since the obvious source of FFA increase is high dietary fat intake and adipose tissue mass, obese subjects will have higher hepatic steatosis compared to those of lean subjects. Accordingly, weight loss achieved through lifestyle modifications (diet and exercise) has been promoted as the standard treatment for NAFLD; particularly due to the lack of other effective treatments [6]. In this study, the body weight and adiposity of HFD fed NAFLD rats were markedly decreased with ETS treatment and the reduction in adipose tissue mass was not simply a reflection of decreased body weight as the decrease in adipose tissue exceeded the body weight changes, suggesting its favourable role in modulation of energy homeostasis.

Several mechanisms can contribute to the accumulation of triglyceride in liver. First of all, it can be sourced from increased hepatic triglyceride synthesis and from redirection of triglyceride metabolism towards storage rather than secretion as VLDL. Secondly, the availability of surplus fatty acids stemmed from uptake of serum FFA, *de novo *lipogenesis in liver, and circulating lipoproteins favour increased hepatic triglyceride synthesis. In addition, fatty-acyl-CoA released by the breakdown of previously stored triglyceride can be re-esterified, though it sans in contributing any new molecule of fatty acid to liver triglyceride pool. Finally, predominance of esterification pathway of fatty acids in the liver, as opposed to metabolic fatty acid oxidation, is also typically associated with an increase in *de novo *lipogenesis. The modifications of these various pathways and their contribution to triglyceride accumulation may be governed by the whole body metabolic status and the stage of progression of steatosis itself, making interpretation of the mechanisms responsible for steatosis, still more challenging [23]. Moreover, sterol regulatory element binding protein-1c (SREBP-1c), a key player in hepatic lipogenesis, activates nearly all genes required for *de novo* synthesis of fatty acid and triglyceride synthesis Overexpression of SREBP-1c enhanced fatty acid synthesis and led to the development of fatty liver on account of increased lipogenesis in transgenic mouse liver and HFD induced rodent models of insulin-resistant diabetes and obesity [24]. In the present study, *T. indica* extract significantly decreased total hepatic lipids and hepatic triglyceride while nonsignificantly decreased hepatic cholesterol, which can be correlated with its ability to modulate multiple pathways narrated above, which controls lipid metabolism in liver especially after HFD induction, albeit the exact mechanism yet to be explored. NAFLD echoes a wide spectrum of fatty liver changes ranging from hepatic steatosis to steatohepatitis (nonalcoholic steatohepatitis, NASH), which can progress to liver fibrosis and cirrhosis in the absence of alcohol consumption [25]. Liver histology of HFD fed NAFLD control rats in the present study revealed typical micro- and macrovesicularsteatosis, hepatocellular ballooning, and vacuolar degeneration, the findings which are in confirmation with previous reports [26]. Meanwhile, histology of the livers of ETS treated rats appeared with less fatty infiltration and hepatic degenerative changes in hepatocytes (Figure 3). Consistent with the alleviation of hepatic steatosis and hepatomegaly by ETS treatment, the activity of hepatic injury marker, such as ALT, also tended to be lower in the sera of extract treated rats suggesting its role in prevention and alleviation of NAFLD in HFD fed rats. ALT is a key enzyme found predominately in the liver. Significantly elevated activity of ALT in serum often suggests its leakage from damaged hepatic cells and reflects hepatocyte damage. Aspartate aminotransferase (AST) is also a hepatic marker enzyme; similar to ALT. However, ALT is a more specific indicator of liver inflammation than AST, as AST may get elevated in diseases affecting other organs as well. For this reason, ALT is commonly used as a way of screening for liver problems, and elevated ALT level is strongly correlated with NAFLD [27]. Therefore, the attenuating effect on ALT by ETS administration in HFD fed NAFLD control rats might account for the improvement of the liver histology and less fatty infiltration in hepatocytes.

According to the “multihit” hypothesis, disrupted lipid metabolism and insulin resistance are the first step towards NAFLD development [4]. FFA appears to be imperative mediators of lipotoxicity, both as potential cellular toxins and by inducing lipid overaccumulation through insulin resistance. It has been demonstrated that a series of molecular alterations in insulin signaling occurs in the setting of insulin resistance, finally resulting in triglyceride accumulation in the liver. The mechanism whereby excessive FFAs in the blood induce insulin resistance is partly through the mediation of protein kinase C, resulting in impaired function of insulin receptor substrate-1 (IRS-1), which further activates janus activating kinase (JNK) and suppressor of cytokine signaling-3 (SOCS3), contributing to insulin resistance. FFA promotes hepatic lipotoxicity also by stimulating TNF expression via a lysosomal pathway, apart from acting as the most important source of reactive oxygen species (ROS) [4, 21].

The index, such as HOMA, provides a reasonable and reliable measure of insulin resistance when applied to rats and mice as it does in humans [28]. Accumulating evidences indicate that insulin resistance contributes to NAFLD directly by increasing *de novo *lipogenesis and indirectly by increasing FFA flux to the liver via decreased inhibition of lipolysis. FFA derived from the lipolysis of visceral and subcutaneous fat account for approximately 60% of the total hepatic FFA content. In addition to increased FFA influx, hyperinsulinemia increases hepatic lipogenesis by activating (SREBP-1c), a key regulator of lipogenic gene expression. Similarly, hyperglycemia caused by inadequate insulin action results in the activation of carbohydrate response element binding protein (CREBP), which activates L-type pyruvate kinase and lipogenic genes in hepatocytes [29].

Hyperglycemia, hyperinsulinemia, and elevated HOMA index associated with HFD administration were restored to normal control levels after ETS treatment at all the three tested dose levels. Likewise, ETS significantly lowered the HFD induced rise in FFA level at all tested dose levels, indicating that the decrement in FFA observed in the treatment groups might have attributed in ameliorating the insulin resistance and improving the insulin signaling by the extracts or vice versa, as reduction of insulin resistance can result in lowering of FFA influx to liver. Recent facts clearly implicate hepatic insulin resistance as the principal factor responsible for the accumulation of FFA as triglycerides in hepatocytes in NAFLD [25]. Therefore, treatment strategies for NAFLD, at present, are primarily focused on weight loss and use of insulin sensitizing agents [6]. In the current study, marked lowering of hepatic triglyceride level as observed with ETS administration can be well substantiated with the reduction in serum FFAs level and further improvement in insulin sensitivity.

NAFLD patients were found to have increased lipid peroxidation, impaired redox balance, and lowered antioxidant capacity. This has led to the idea that dietary antioxidant supplements may protect against the damaging effects of the disease. Infiltration of fat in hepatocytes as observed in NAFLD has been reported to elevate the rate of *β*-oxidation resulting in excessive production of ROS, which ultimately may give rise to abnormalities in mitochondrial morphology and their dysfunction. This sequence of events snowballs into a chain reaction resulting in damage to mitochondrial membrane [27]. In addition, excessive FFA that induces insulin resistance can also trigger the release of ROS and proinflammatory mediators from impaired liver cell organelles, thus contributing to the subsequent “hits” in the progression of NAFLD. Lipid peroxidation of microsome is upregulated and *β*-oxidation of mitochondrion is downregulated by insulin resistance, which make the liver more sensitive to oxidative stress. Lipid peroxidation further leads to the generation of by-products, such as malondialdehyde (MDA), which are involved in activation of the inflammatory response and, consequently, cellular damage [4]. The present findings of high hepatic MDA levels in the steatosis group or NAFLD control group are in sound agreement with the previous reports [21, 30] and the oral administration of ETS extract was associated with lowering of MDA content in liver significantly. It is expected that the potential effect of ETS in preventing further accumulation of free radicals as well as oxidative stress could be due to the ability to reduce free radical formation or through the free radical scavenging activity. Moreover, the previous reports on tamarind seed coat extract have specified increase in the antioxidant enzymes and a decrease in the oxidative stress [9, 10]which is in confirmation with present findings.

Scientific evidences indicating the presence of numerous polyphenols including tannins, anthocyanidin, and oligomeric anthocyanidins in *T. indica* seed coat extract are already available [11]. Polyphenols are affirmed to suppress lipogenesis and have signposted to have fatty acid synthase inhibitory activity *in vitro *while tannic acid specifically is testified for its potential to inhibit adipogenesis [7, 31]. Although further qualitative and quantitative phytochemicals analyses of tamarindus seed coat are necessary, the present results suggest that the ameliorative effect of ETS on hepatic steatosis, partly mediated via antiobesity, insulin sensitizing, and/or antioxidant mechanisms in HFD fed rat model of NAFLD may be attributable to these phytochemicals. Overall, the role of ETS extract as a potential herbal therapeutic agent against NAFLD as indicated in this study warrants further investigation in order to delineate the underlying mechanisms and to identify the germane antifatty liver phyto-principles in ETS for plausible clinical use.

In conclusion, the present study, for the first time, shows the ameliorative potential of *Tamarindus indica* seed coat on HFD induced NAFLD in albino Wistar rats that may partly be correlated with its antiobesity, insulin sensitizing, and antioxidant mechanisms. These results, thus, provide insights into the clinical therapeutic potential of ETS in management of NAFLD that can be further explored as a new therapeutic for NAFLD.

## Highlights

- High fat diet (HFD) fed rat model with clinical features of NAFLD is validated;
- Extract of *Tamarindus indica* (ETS) is evaluated in NAFLD rat model;
- ETS alleviated HFD induced high hepatic lipids, lipid peroxides, and hepatomegaly;
- ETS attenuated HFD induced insulin resistance and raised FFA levels;
- ETS mitigated HFD induced obesity and adiposity.

## Figures and Tables

**Table 1:** Composition of experimental diets.

Formula	Normal Diet		HFD-45% kcal	
	g%	kcal%	g%	kcal%
Protein	20.3	21.3	25.4	21.3
Carbohydrate	68.1	71.6	40.0	33.7
Fat	3.0	7.1	24.0	45.0
Total		**100**		**100**
kcal/g	3.8		4.8	

Ingredients	Normal Diet		HFD-45% kcal	
	g	kcal	g	kcal

Casein	200	800	250.1	1000
L-Cystine	3	12	3.75	15
Soybean oil	30	270	37.5	338
Lard	0	0	200.3	1803
Cornstarch	438.59	1754	97.65	391
Maltodextrin	132	528	165.1	660
Sucrose	100	400	125	500
AIN-93 vitamin mix	10	40	12.5	50
AIN-93G mineral mix	35	0	43.8	0
Choline chloride	1.40	0	1.75	0
Cellulose	50	0	62.5	0
t-BHQ	0.01	0	0.05	0

Total	1000	3804	1000	4757

Diets were prepared according to the AIN-93G diet composition with slight modifications. Mineral mixture and vitamin mixture were as per AIN-93G recommendations.

AIN: American Institute of Nutrition; HFD: high fat diet, t-BHQ: Tert-butyl hydroquinone.

**Table 2:** Effect of ETS treatment for six weeks on terminal serum and hepatic parameters in HFD-fed rat model of NAFLD.

Parameters	I ND control	II NAFLD control	III HFD + ETS at 45 mg/kg	IV HFD + ETS at 90 mg/kg	V HFD + ETS at 180 mg/kg
Liver index	2.43 ± 0.04	2.89 ± 0.12^a^	2.25 ± 0.08^b^	2.34 ± 0.07^b^	2.29 ± 0.06^b^
MDA (nmol/g liver tissue)	17.91 ± 1.70	32.26 ± 4.03^a^	13.90 ± 1.04^b^	14.06 ± 1.17^b^	14.53 ± 1.36^b^
Serum ALT (IU/L)	34.89 ± 1.84	59.52 ± 3.45^a^	28.28 ± 0.79^b^	27.59 ± 2.41^b^	28.28 ± 2.38^b^
Serum free fatty acids (mEq/L)	0.86 ± 0.06	2.04 ± 0.25^a^	0.72 ± 0.06^b^	0.69 ± 0.09^b^	0.74 ± 0.11^b^
Serum Insulin (µIU/mL)	18.65 ± 1.37	29.68 ± 3.21^a^	23.34 ± 2.08	28.77 ± 2.38	26.20 ± 2.67
Serum glucose (mmol/L)	3.94 ± 0.31	4.98 ± 0.33	4.31 ± 0.16	3.92 ± 0.29	3.78 ± 0.15^b^
HOMA	3.23 ± 0.24	6.59 ± 0.81^a^	4.44 ± 0.34	4.94 ± 0.37	4.36 ± 0.41

Values are expressed as Mean ± SEM; *n* = 6; ^a^ *P* ≤ 0.05, NAFLD control versus ND control; ^b^ *P* ≤ 0.05, treated groups versus NAFLD control.

Liver index = (absolute liver weight/Body weight) ∗ 100.

ETS: extract of *Tamarindus indica *seed coat; NAFLD: non-alcoholic fatty liver disease; ND: normal diet; HFD: high fat diet; MDA: malondialdehyde; ALT: alanine aminotransferase; HOMA: homeostatic model assessment.

## Abbreviations

NAFLD: Nonalcoholic fatty liver disease
ETS: Extract of *Tamarindus indica* seed coat
NASH: Nonalcoholic steatohepatitis
ROS: Reactive oxygen species
ND: Normal diet
HFD: High fat diet
NISCAIR: National Institute of Science Communication and Information Resources
FFA: Free fatty acids
AST: Aspartate aminotransferase
ALT: Alanine amino transferase
MDA: Malondialdehyde
VLDL: Very low density lipoproteins
H&E: Hematoxylin and eosin
LPO: Lipid peroxidation
HTRF: Homogeneous time resolved fluorescence
HOMA: Homeostatic model assessment
SREBP-1c: Sterol regulatory element binding protein-1c
IRS-1: Insulin receptor substrate-1
JAK: Janus activating kinase
SOCS-3: Suppressor of cytokine signaling-3
CREBP: Carbohydrate response element binding protein.
